# Correspondence

**Published:** 1874-08

**Authors:** 

**Affiliations:** Chillicothe


### Correspondence,

PROF. J. TAFT:

*Dear Sir*, In answer to your inquiry as to the results of my experimental use of Lacto-Phosphate of Lime as a local application to exposed dental pulp, sensitive and softened dentine, I can only reiterate and reaffirm everything which I have from time to time said to you in conversation on this subject. My faith in the correctness of the theory and efficacy of the agent is constantly increasing, and I have no doubt, as soon as it is more generally used, *not abused*, that it will rank as one of the most valuable of local applications for the production of specific effects, which has presented itself within the last decade.

Having for the last five or six years eagerly availed myself of everything offered or suggested as a rational agent for the protection of an exposed pulp, or for its restoration to health when diseased, I have of course tried many things; some of them upon the high recommendation of professional brethren upon whose judgment I relied, in preference to my own, notwithstanding that in point of theory I differed widely from them.

For instance, distrusting my own convictions, I under protest, as it were, experimented with oxychloride of zinc, for the protection of exposed pulp tissue, for I could not reconcile it with reason that the chloride of zinc, in strength unknown, said strength being regulated by the manufacturer solely with a view to expedite the setting of the cement and which habitually acts upon all animal tissue as an escharotic, should act beneficially or *even* harmlessly upon the delicate and highly vascular substance we speak of.

Chloride of zinc must be valuable to us as an escharotice, owing to its rapid destruction of morbid growths, etc., but this very property which it most surely possesses, should at once preclude all idea of using it as a nerve covering.

It is curious to look over the proceedings of societies, say of three or four years back, and read reports of wonderful success in saving teeth with this agent, and call to mind its general laudation for this purpose.

Well, it is now thought best, to protect the pulp with something else, say the lining membrane of an egg shell, gold beaters skin, etc., etc.

This repudiation of oxychloride of zinc as a *direct* and immediate application to pulp tissue should be gladly hailed by the public at large, as they are most nearly interested, as for ourselves, I feel that we have newly discovered a lost pathway, are again in the right track.

I found in the oxide of zinc a more genial friend to an exposed and sensitive pulp, and have used it experimentally, mixed with plaster paris, sufficient to give it setting qualities. Felt greatly satisfied with the result, and when Dr. King, of Nashville, about two years since, called the attention of the Profession to this very thing, I felt that a large step had been taken towards the lost path.

Having for years firmly believed, that a pulp recently exposed and comparatively healthy, is, in most cases, "doctored to death" and that the simplest and mildest treatment offers the best chance for the preservation of its vitality, I was prepared to hail with delight the introduction of the Lacto-Phosphate of Lime.

What have we in this preparation? We have in the first place a perfectly harmless, pain-relieving application for sensitive and softened dentine. Cases which now call for the application of nitric acid, or argt. nitric. Cases in which the lime salts are rapidly dissolved, leaving the animel frame work standing, and presenting that well known gelatinous condition of dentine, will, under reasonably favorable accompanying circumstances of constitution, harden and fill up with new deposits of lime salts, often after the second, third or fourth application.

Were this the end of its efficacy, would it not be a valuable addition to our materia medica?

But this is not all, it is one of the best applications I have ever met with, for sensitive dentine, that sensitiveness, which can not well be overcome by rapid strokes or cuts of a sharp excavator. As a covering for an exposed tooth pulp it is invaluable, causing no pain in its application, rather subduing it, if any be present, and, if the directions for its use, as set forth by Dr. J. E. Cravans, of Missouri, in the September No. 1873, of *Missouri Dental Journal* page 347, are strictly followed, the result will surely satisfy any one whose expectations are not unreasonable.

I do not wish to assert that this agent is a "sure shot" in the hands of all who may choose to use it. It requires care and skill in its application, and time and patience, to properly prepare a tooth for filling. Let him, therefore, who expects to get his tooth in order, fill it, and, *pocket his fee, all in one sitting*, pass it by; it will be "too slow" for him.

But those of our brethren who have a real interest in the welfare of their fellow men, together with an earnest desire for the advancement of dental science, will here have an opportunity of testing a beautiful theory, and with the hope of a most satisfactory issue.

I am truly glad that you have succeeded in having some of the Lacto-Phosphate of Lime prepared in Cincinnati. The makers name, Prof. Vaughn, is a surety of a first rate article. Induce all your friends to give it an impartial, fair and just trial, and ask them to report results.

Before I close, I ought to caution those who may wish to try it, to be as careful in its application, to keep the cavity as dry, as if they were preparing to fill the tooth with cohesive gold, always applying the rubber dam. Any mixture of the saliva with the paste injuring, if not destroying its efficacy.

You have my last correspondence with Dr. Cravans on this subject, this you may publish in full, if you like. To the gentleman just named, the profession is indebted as the originator of this method of treatment of exposed pulps, and that his theory may prove correct and its practice be the means of saving the vitality of many thousands of teeth is the sincere hope and wish of your friend, REHWINKEL.

CHILLICOTHE, June 24, 1874.

